# Long-Term Engagement in Formal Volunteering and Well-Being: An Exploratory Indian Study

**DOI:** 10.3390/bs6040020

**Published:** 2016-09-27

**Authors:** Jereesh K. Elias, Paulomi Sudhir, Seema Mehrotra

**Affiliations:** Department of Clinical Psychology, National Institute of Mental Health and Neuro Sciences, NIMHANS, Bangalore 560029, India; jereeshelias@gmail.com (J.K.E.); paulomi@nimhans.ac.in (P.S.)

**Keywords:** volunteering, formal volunteering, volunteering and well-being, well-being, psychological well-being

## Abstract

Sustained engagement in volunteering and its correlates have been examined in many studies across the globe. However, there is a dearth of research that explores the perspectives of long-term formal volunteers on the nature of changes perceived in oneself as a result of volunteering. Moreover, the linkages between psychological well-being and volunteering have been insufficiently explored. The present study was aimed at addressing these gaps. A heterogeneous sample of 20 long-term formal volunteer engaged in volunteering across different voluntary organisations in a southern metropolitan Indian city formed the primary sample for the study. In addition, a group of 21 short-term volunteers, matched on age, income and gender, was utilised for comparison with long-term volunteers on well-being indices. A semi structured interview schedule was used to explore self-perceived changes attributable to volunteering experience. In addition, a few standardised measures were used to comprehensively assess subjective well-being and psychological well-being. The interview data provided rich descriptions of perceived positive changes in self across cognitive, behavioral and emotional domains. Mirroring these patterns, the quantitative analyses indicated that long-term volunteers experienced higher levels of psychological well-being (sense of mastery and competence, self-acceptance and sense of engagement and growth) than short-term volunteers. The potential mechanisms involved in beneficial outcomes of long-term volunteering and implications for further research are highlighted.

## 1. Introduction

### Volunteering Research

The study of volunteering has attracted the attention of scientists from varied disciplines such as philosophy, economics and sociology to psychology and public health. Volunteering has been defined as “any activity which involves spending time, unpaid, doing something which aims to benefit someone (individuals or groups) other than or in addition to close relatives, or to benefit the environment” [1]. Formal volunteering refers to “unpaid help given as part of groups, clubs or organizations to benefit others or the environment” [2]. It includes devoting time and effort on a planned basis primarily for the benefit of others, the community or environment [3].

In India, the early forms of charity and voluntary activities were often carried out as part of religious practices or in religious contexts. The Indian concept of “Shramdaan” (donating labor/efforts) comes very close to the concept of volunteerism; while there are other concepts which are related more to charity [4,5]. The tradition of volunteering has existed in India for a long time although formal volunteering through organizations became more popular only in the post-independence period, especially in the 1980s [6]. With the steady rise in the avenues for formal volunteering, it becomes important to examine volunteering and its mental health correlates, especially as global research and observations suggest that volunteering can positively influence not only the direct recipients of volunteering activities and the society at large but may also benefit the volunteers themselves [7,8].

## 2. Literature Review

### 2.1. Benefits of Volunteering

Volunteering has been associated with a range of positive psychological outcomes for the volunteers [9,10]. Positive outcomes are being examined not just in terms of mental health and general physical health but also in terms of strengthening social connections and thereby resulting in effective social integration [11].

Thoits and Hewit [12] observed in their longitudinal study that volunteer-hours were associated with significantly increased happiness, life satisfaction, mastery and physical health. It was observed that volunteering and well-being positively and reciprocally influenced each other. Meier and Stutzer [13] utilizing a large scale survey data from the German socio economic panel also observed a reciprocal relationship between volunteering and life satisfaction. Using data from three waves of the Americans’ Changing Lives Study, Li and Ferraro [11] found that volunteering contributed to better mental health in the elderly and was linked to a lower pace of functional decline, higher social engagement and a buffering impact against depressive symptoms. A review by Swinson [10] on volunteerism indicated beneficial influence on anxiety, depression, self-esteem and physical health. Dass-Brailsford, Thomley and de Mendoza [14] documented transformative effects of volunteerism using the experience of volunteers who engaged in the relief activities of Hurricane Katrina in US. To understand the potential utility of volunteering as a public health intervention, Jenkinson et al. [15] conducted a meta-analytic study. They noted that reduction in depression, improvement in life satisfaction and well-being and reduced mortality rate among volunteers were seen in cohort studies. Shen, Pickard and Johnson [16] tried to unravel the utility of volunteering for depression and self-esteem in a sample of African American females who were primary caregivers for older adults above age 65 years. An examination of the British household panel survey data utilizing self-report of engagement in volunteering and the General Health Questionnaire (GHQ) revealed that regular engagement in volunteering was associated with higher levels of well-being [17]. This study also revealed life-course variability in terms of its observation that the association between volunteering and well-being emerged only from age 40 onward. Yet, another study utilizing a British Household Panel Survey data set demonstrated a positive impact of volunteering on well-being. Also, it was noted that sustained engagement in volunteering increased its positive impact over time. This pattern highlighted that the beneficial effects of volunteering on well-being may not be subject to hedonic adaptation [18]. Prolonged exposure to volunteering has been associated with beneficial outcomes in other studies, too [19].

### 2.2. Volunteering Benefits in the Context of Theory and Research on Well-Being

Although well-being outcomes of volunteering have been examined in the field of volunteering research, these have not been consistently linked to existing theoretical and research literature on well-being. In the available research literature on well-being, engagement in different kinds of meaningful activities has been associated with a variety of positive outcomes. Different types of behavioral activities, such as trying to be kind to others [20] as well as cognitive activities, such as reframing situations in a more positive light, counting one’s blessings [21,22] and volitional activities, such as investing efforts in meaningful causes [23] have been associated with well-being. It is also known that accumulations of daily experiences that satisfy needs such as competence, relatedness, and autonomy can lead to enhanced global well-being [24]. Such studies suggest that volunteering may enhance well-being through various means, for example, through providing opportunities for meaningful engagement, development of a sense of gratitude through comparison with those in more disadvantaged contexts, through satisfaction of needs for competence and relatedness and through an enhanced sense of meaning in life, etc. In the youth-studies literature too, engagement in meaningful activities, beyond a narrow focus on self is seen as contributing to positive youth development characterized by the five Cs, namely competence, confidence, caring, connection, and character [25]. Intentional activities have also been hypothesized to play an important role in achieving sustained levels of well-being [26]. Intentional activities can result in a diverse array of experiences as well as generate new opportunities and possibilities. Activity-changes have also been documented to result in enhanced psychological well-being, presumably through their impact on sense of mastery, relatedness, etc. [27]. Similarly, Peterson, Park and Seligman [28] proposed pleasure, meaning and engagement as three pathways to happiness and demonstrated that these pathways were associated with life satisfaction. These observations again point to the potential relevance of sustained engagement in volunteering for maintaining/enhancing well-being.

### 2.3. Indian Research Related to Volunteering

There are a few Indian studies on altruism and its correlates especially in children and adolescents [29,30]. Bhangaokar and Mehta [31] examined youth civic engagement in the Indian context. In-depth interviews were conducted with purposively sampled 19 civically engaged youth. Findings revealed that increased perspective-taking, identification with and feeling responsible for the in-group led to a broadening of focus on social issues. Changed perspective/ideological transformation, skill building, and comfort with identity were some of the positive changes reported in this study. In an evaluative study conducted by Tata Institute of Social Sciences (2008–2009) on the National Service Scheme (NSS for the Ministry of Youth Affairs & Sports, Government of India), 723 NSS volunteers were sampled from different parts of India. Increased confidence, communication skills and awareness of social issues were reported by the participants as some of the outcomes of volunteering [32].

### 2.4. Rationale for the Study and Research Questions

On the whole, psychological outcomes of formal volunteering have been examined across the globe. However, most studies have focused on a restricted range of well-being outcomes. Beneficial impact on variables such as depression, anxiety, self-esteem and life-satisfaction have been most frequently examined. Psychological well-being and its dimensions such as a sense of environmental mastery, self-acceptance, sense of purpose etc. have been rarely examined in this context. Global research on psychological outcomes of volunteering has often focused on older adults. Most of the studies have made use of quantitative methods, which leave little scope for a richer understanding of the psychological processes that may co-occur with exposure to volunteering. Moreover, there are very few studies that shed light on whether long-term volunteers perceive personal changes as a result of volunteering experience and the nature of any such changes. The subjective accounts of volunteers are likely to help in better understanding the ways in which volunteering might have a beneficial impact on their well-being. These observations highlight the need for qualitative studies in the field of volunteering research. There is a significant paucity of Indian research even in the broader field of formal volunteering and its mental health/well-being correlates. The few Indian studies available so far have mostly involved student-samples.

In the above context, the present study was carried out to address the following research questions:

- What is the nature of changes, if any, that long-term volunteers perceive in themselves and attribute to engagement in volunteering?
- What are the levels of well-being that the long-term volunteers report on standardized measures of subjective and psychological well-being?
- How do well-being scores of long-term volunteers compare with those of a matched group of volunteers who are engaged in volunteering for a short period?

This paper is based on part of a larger study on understanding determinants, process and mental health outcomes of formal volunteering. Existing research suggests that the beneficial impact of volunteering increases over time with sustained engagement in volunteering. Moreover, changes in psychological well-being are likely to take longer than changes in subjective/emotional well-being. These issues can be examined well in studies using longitudinal designs. However, such a design was not feasible in the present study context. Hence, it was planned to compare the long-term volunteer sample to a short-term volunteer sample because intention to volunteer and actual initiation of volunteering can be considered as their common attributes which enhance their comparability.

## 3. Method

### 3.1. Design of the study 

A cross sectional, mixed methods design involving qualitative and quantitative methods was used.

### 3.2. Tools Used

Subjective well-being: Subjective well-being was assessed in terms of affective and cognitive components as mentioned below.

Positive and Negative Affect Schedule (PANAS-Revised) was used to assess affect in the last few weeks. It was developed by Watson, Clark and Tellegen [33] and an expansion to include a wider range of affect items was suggested by Barrett and Russell [34] in 1998. Accordingly, the phrasings were modified and six items were added and adapted for use in Indian samples by Rao and Mehrotra [35]. The measure has sound psychometric properties as examined in an Indian study involving a large community sample [36].

Satisfaction with Life Scale (SWLS) [37]: The SWLS is a short 5-items scale designed to measure global cognitive judgments of one’s life. The internal consistency reliability and test-retest reliability as well as construct validity data from various samples indicate adequate psychometric properties in studies across the globe, including in India [36].

Psychological Well-being: This was planned to be assessed through the use of a psychological well-being measure (PWB-20) [38]. It is a 20 item measure derived from Ryff’s conceptualization of psychological well-being [39] and has been derived based on pilot testing in Indian samples. It consists of the following factor-based subscales with satisfactory reliability and validity: sense of engagement and growth, positive relationships with others, self-acceptance and sense of mastery and competence.

Semi-structured Interview Schedule: Interview probes were developed for use with long-term volunteers. The probes aimed at exploring experiences of volunteering, motives for and ways of sustaining volunteering, facilitators and barriers, as well as self-perceived changes in oneself in terms of one’s personal beliefs and behaviors as a result of volunteering experiences, etc. An exploration of all these aspects formed a part of a larger study on volunteering and mental health. The present paper is restricted to exploring self-reported changes as attributed by the participants to engagement in formal volunteering. The interview probes that are relevant for this paper included: “What have been your experiences, in general, during volunteering? What makes your volunteering satisfying/dissatisfying? Has the experience of volunteering influenced you in any way as a person? If so, please describe.”

### 3.3. Sample

In the present study, a long-term volunteer was defined as a formal volunteer who had been volunteering in one/more organization regularly (at least for one hour, on at least once a month basis) for a period of at least one year. A break from volunteering for a maximum of two months over a one year period, due to external exigencies (such as major life changes), did not result in exclusion from this category in the present study. Similarly, a short-term volunteer was defined as a formal volunteer who had been volunteering in one/more organization regularly (at least for one hour, on at least once a month basis) for a period of six months. The study was carried out in a metropolitan city in Southern India. The sampling was purposive in nature. An updated and comprehensive local directory of Non-governmental organizations (NGOs) was not available at the time of initiation of the present study. Two online sources were used to develop a list of NGOs. This resulted in a directory of 246 NGOs. These NGOs were contacted either by a visit in-person and/or over phone or e-mails resulting in identification of 137 functional NGOs. Further filtering according to the availability of volunteers reduced the list to 97 NGOs. These NGOs were involved in various causes and their identified foci of activities ranged from children’s issues, disability, education, health, gender, senior citizens to environmental issues. In addition, about one fourth of the NGOs were involved in multiple social causes. It was decided to attempt to recruit volunteers from NGOs with varied foci of activities. The study was conducted after seeking ethical clearance from the Institute Ethics Committee.

Twenty long-term volunteers as operationally defined above were recruited from 16 different NGOs. Only those long-term volunteers who were 18 years or older and who could read, write and speak in English were included in the sample. Long-term volunteers who met sample-selection criteria were contacted over the phone and explained about the study and the procedure for collection of data. Those volunteers who were willing to participate and provided written informed consent were met at a mutually convenient place and time. The recruitment of short-term volunteers involved the same sample selection criteria and process except that it entailed recruiting those who were engaged in formal volunteering for a duration of six months.

### 3.4. Method of Data Collection

After obtaining the written informed consent, the data collection was done on a one to one basis, either in a single session of one and half hour duration or two sessions of about 45 min each. The entire data collection process for the larger study was carried out from February 2012 till October 2013 by the first author under the supervision of the second and the third author. The interviews were audio-taped after taking the consent from the participants. A survey proforma containing basic data sheet and questionnaires was administered after the interview, either in the same session or in the second session, according to the convenience of the participant. Data collection with the short-term volunteers required administration of questionnaires on a one to one basis.

### 3.5. Analyses

Normality of the data was examined through Kolmogorov-Smirnov Z test so as to apply parametric or non-parametric tests as appropriate. Interviews were audio-taped and transcribed. Short-term and long term volunteer samples were compared using an independent samples t-test and a Mann Whitney U test. Content analysis was carried out for responses to the questions in the interview. Sandelowski and Barroso [40] stated that research approaches can be placed on a continuum based on the degree of transformation of data during the data analysis process from description to interpretation. Content analysis lies towards the descriptive end of this continuum as it primarily describes the data and/or interprets its manifest content. The procedure used for content analysis was as described by Vaismoradi, Turunen and Bondas [41]. Emergent themes in the responses were identified and a frequency count was carried out. The extraction of themes was carried out by the first author. The third author examined a quarter of the randomly selected transcripts to arrive at the codes independently. As the analysis was carried out at the descriptive level (rather than involving deeper interpretations more typical of thematic analysis), there were very minor discrepancies between coders and these were resolved through consensus. The pooling of themes to arrive at categories was carried out jointly by the authors. The narratives/excerpts of the narratives were extensively documented to retain the richness of qualitative data and provide support for the inferences made.

## 4. Results

### 4.1. Basic Profile of Long-Term Volunteers

The long-term volunteers sampled in the present study were within 21–50 years of age and an average long-term volunteer was 32 years old. Almost two thirds (*n* = 13) of the long-term volunteers sampled were young adults (18–35 years). The remaining were middle aged (*n* = 7). About two third of long-term volunteers were undergraduates (15 out of 20). The remaining had higher educational qualifications. A majority (*n* = 16) were Hindu by religion. Single and married volunteers were equally represented in the long-term volunteers’ sample. There was a slight preponderance of women long-term volunteers compared to men (*n* = 11 and 9, respectively). Majority of the long-term volunteers were engaged in full-time paid work (*n* = 9). A quarter of the sample consisted of home makers (*n* = 5). Students, self-employed persons and those with part-time employment were also represented to a small extent in the sample. Two thirds of the sample (*n* = 14) reported themselves to be fairly well off or very well off in terms of the adequacy of financial resources to meet their day to day demands, while four reported that they were managing with some difficulty or were barely able to manage their day-to-day demands. None of the volunteers reported having any major physical illness, in the last 6 months. Two had sought treatment for a psychiatric/psychological problem in the last 6 months. About two thirds of the volunteers (*n* = 13) had initiated volunteering for a social cause between 18 and 35 years of age. There was a small proportion of long-term volunteers (*n* = 5) who had started formal volunteering during childhood/early adolescence. The average age at which the long-term volunteers initiated volunteering for a social cause was 23 years.

Half of the long-term volunteers sample was engaged in formal volunteering for the past 3–5 years. One fourth of the long-term volunteers sample reported that they were engaged in volunteering for more than 5 years (Maximum = 30 years). Another five volunteers reported of engaging in volunteering for the past 2 years. Almost half of the long-term volunteers (*n* = 9) were engaged in formal volunteering on a more than once-a-week basis. A small number of participants (*n* = 3) were engaged in volunteering on a daily basis in the last 1 year period. Others (*n* = 8) reported engagement in volunteering on once a week/once in a 2–4 week basis. Half of the volunteers (*n* = 10) reported spending more than 8 h/month on formal volunteering. A quarter of the sample (*n* = 5) were spending 4–8 h/month, while another quarter (*n* = 5) were spending 1–4 h/month on formal volunteering. Most of the long-term volunteers were involved in multiple kinds of volunteering activities.

### 4.2. Perceived Changes in Long-Term Volunteers

In response to a few open-ended questions in the interviews, all the long-term volunteers described one/more ways in which they had changed as a result of volunteering experiences. The data could be coded by examining them from different perspectives. While labeling the emergent themes and pooling them into broad categories, the researchers attempted to stay close to what was explicitly emphasized/the aspect that was most focused upon by the participants, Although a few of the emergent themes were similar to the dimensions of psychological and social wellbeing, utilization of these for coding would have required deeper level interpretations in some instances. For example, increased self-regulation might contribute to a sense of mastery and a sense of personal growth, but these were not explicitly mentioned as such by the participants. It was decided to use the broad categories of cognitive, affective and behavioral changes for the following reasons: (a) These highlighted the different levels at which changes were reported by the participants; (b) It was possible to group themes into these broad categories without deeper level inferences; (c) These three categories are common categories to describe various kinds of psychological data including coping, changes in self, etc. Though these are certainly interlinked and overlapping, it was possible to use the following rules of thumb in the process of categorizing.

Any theme which entailed description of specific changes in one’s behavior was placed under the behavioral change category. Themes explicitly referring to changes in one’s patterns of thinking/perceiving/beliefs or values were placed under the cognitive category. Changes in nature of emotional experiences as well as regulation of emotional experiences were placed in the emotional changes category. This included instances wherein the focus of the participants’ narratives was on highlighting changes in emotional experiences, sometimes due to better regulation. It may be noted that these categories are not completely non-overlapping and also that the participants often reported more than one kind of perceived change in themselves. The statements were coded under a given category based on the researcher’s understanding of the main thrust of the participant’s responses although changes at one level are likely to co-occur with/result from changes at another level (e.g., decreased self-centeredness as self-reported is likely to result in increase in compassionate behaviors. This would be an instance of cognitive change co-occurring with behavioral change. The various themes that emerged under these domains (Table 1) are enumerated below along with a few examples.

#### 4.2.1. Cognitive Changes

*Decreased self-centeredness:* Some long-term volunteers expressed that they were able to look beyond their own needs and understand others’ needs, as a result of their engagement in volunteering. They also reported of being able to assign more importance to others’ needs whenever needed *(e.g., “…it’s so easy to live a self-centered life. It’s so easy. I mean from the time I was a kid everything and everybody was for me. So I became very entitled. You know you become very self-centered and you expect the same thing in the school, your friends to do the same thing to you, your teachers…These things will get moderated. That’s why I owe volunteering so much, because it made me a better person.”; “I have become more selfless. My selfish nature has gone. Only for me…only for me…that character has gone. Even people who mingle with me also tell there are changes. My mom use to say I have changed positively.”).*

*Inculcation/strengthening of specific values:* Some of the examples of the values inculcated through engagement in volunteering, as reported by the participants were active citizenship, being non-judgmental, and sharing as well as a reduced focus on materialistic gains/pursuits, etc. *(“…so this being socially aware, civic awareness has become part of me now. So as I said even if I have to take a break from volunteering, I won’t take a break from the values which I have inculcated. Unconsciously also, we spread this awareness to whoever we meet.”; “And tell myself that wait…people are not as bad as I think. That judging people on the face has changed”; “I gained this knowledge and I didn’t want to keep this to myself. So someone who is close to me or in the surrounding, I share my experiences. That’s where I am transforming myself about seeing things in a different way.”).*

*Enhanced awareness of repercussions of behaviours:* To take an example to illustrate this change, a long-term volunteer reported that as a result of engagement in volunteering activities, she started thinking about the repercussions of current behaviours on the future generations *(“Through volunteering I came to know that what we do wrong, it’s going to affect our city later or the locality. Here the knowledge I gained is important. Sometimes, some people will tell, how does it matter if it’s just one piece of paper or plastic? But I could tell them with authority, how one plastic bag can affect the entire locality and things like that”).*

*Changed perspective about minor personal issues:* During the engagement in volunteering one volunteer witnessed the life-struggles that some people go through, which she referred to as “*I could see the raw life there*”. According to her, witnessing such a life resulted in having insight/brought a change in the perception about minor personal issues in day to day life *(“We visited one organization where the children who engaged in some offense at a very young age are kept. I saw how they are trying to bring these children to the mainstream. The talents the children have got...I got all these experiences and exposures. That naturally enriched me and influenced how I saw the society in the beginning and now. Like in my family, all have done PhDs or M. SCs and they are working in MNCs, and we protect the children very much...give whatever they ask. I witnessed a different kind of life directly, I saw these children, I talked to them. It made me think deeply about their status. What these children are getting? And what my relatives’ children are getting? It really created an insight, that we fight for trivial things in our homes, like if husband doesn’t come home on time, or if children argue with us, we say “I am depressed today”).*

*Gaining a sense of purpose in life/clarity in life goals:* Some long-term volunteers reported that engaging in volunteering gave them a sense of purpose in their life *(“…Definitely, before I used to think why to live? Before coming here, that suicidal thinking was there. Whenever my energy goes down—Ok I will stop it, I will go somewhere else—like that I used to think. Now I stopped thinking like that.”; “Actually I got my life from volunteering (This long-term volunteer had undergone treatment for depressive disorder earlier and was prompted by the counsellor to engage in volunteering). Volunteering is like my parent. Without volunteering, there is no life for me”). Also, some of them reported of gaining clarity about their goals in life by engaging in volunteering (“I have more clarity about what I want to do with my life”).*

*Heightened awareness of environment:* Some long-term volunteers reported that their style of “living, without much awareness of surroundings” has changed *(“Even if they are in front of your eyes you won’t see them. But now that I am teaching children, I teach children civic sense. Now that when I go to street and I look at what I teach my children…keeping the street clean and not littering and knowing your rights and responsibilities… now I have started noticing it…which I did not notice earlier.”).*

*Increased tolerance and empathy:* Some of the long-term volunteers reported that before initiating volunteering, they harbored prejudices about people from different social class. They perceived that as a result of engagement in volunteering, their prejudices had diminished and they were able to interact well with people from a lower social-class than theirs *(“Even low class people—I should not call them like that—but earlier I had hesitation but now everybody is approachable and I can go and interact with them. I won’t say I changed fully but there is a good change in my attitude towards them. I think from their perspective also…What if I was in their position.”; “Yes my prejudices about people in village or slum have gone. I thought they are people who will create problems for urban people. They may not accept us. But it all changed.”; “I spend 100 Rs. and I will think what that 100 Rs. means to my child [the child she is mentoring]. So that has been a change.”).*

Change in the meaning of giving: *“Our parents started asking why giving things and all. That time we realized that commodities are not the only thing which we can give. Why can’t we sit with a security guard and have a good conversation with him. That is also social service; we came to know there are different forms of social service. This makes them smile, this makes them laugh.”*

*Willingness to go beyond comfort* *zones (Openness):“Earlier I was keen about where I work, how much I work. I can only work for 9 h in Bangalore. But then I saw people, disabled people who are ready to work anywhere in the world. If they can why can’t I? I am at least abler than them. It has changed my perspective. I think maybe I had taken things for granted.”*

#### 4.2.2. Behavioral Changes

*Reduction in problem behaviors and interpersonal conflicts:* Some long-term volunteers reported that their aggressive tendency had been reduced as a result of engagement in volunteering *(“Earlier I was into gang fights and all. After I started helping people, that tendency of aggression has come down.”).* A change in the quality of interpersonal relationships was evidenced as a reduction in interpersonal conflicts *(“Even when you fight with your spouse, you can see how much you are centered on yourself. These things get moderated. It reduced fights between us. That’s why I owe volunteering so much.”).*

*Increased assertiveness as a citizen:* A few long-term volunteers reported that they had gained the knowledge and skills to assert their rights as a citizen *(“Earlier I didn’t know what are the rights or responsibilities of a citizen. I knew people should keep the street clean, but I did not know how to tell them. CMCA taught me ways to channelize my thoughts and about being a little more firm when I tell people…And when they say ‘mind your business’ I can tell them—‘yes it is my business. I want my city to be clean. I have paid tax I can’t dirty my city’. Then people understand.”; “Standing up for my own rights, being responsible it’s kind of become part of me now. That has become a habit for me now.”).*

*Other changes in social behaviors*: Some of the long-term volunteers reported that engaging in volunteering helped them to become more confident in social situations *(“Though I have studied engineering, I was ignorant about many things. Now I can go and talk to people, it gave me confidence”; “So we can mingle with any group. We know how much we can talk, which are all the areas we can talk about and what to avoid discussing about. All these things we know.”; “I have done my under graduation course in management but I could not speak confidently to a mass. But now I am able to address ‘n’ number of people, whether it is adults, youth or any population.”).* One of the volunteers reported of becoming more polite and respectful in interpersonal interactions with others *(“I respect people more and have become more polite.”).*

*Practice of civic duties in day to day life:* Some of the long-term volunteers reported that they started practicing what they teach *(“Unconsciously also, we spread this awareness to whoever we meet. Like yesterday, when I was going in the auto, he [auto-driver] was constantly spitting. So I couldn’t stop myself from telling [laughing] I told him ‘please stop spitting’. I don’t just say how things should be—I do things that way.”).*

*Increased self-regulation and focus and persistence in one’s goal pursuit***:** One long-term volunteer felt that engagement in volunteering gave her clarity about life goals and she was able to be persistent and focused in its pursuit *(“I am totally focused and determined and I like the work I do and I am engrossed in the work” Developmental Economics is the favorite subject of this particular long-term volunteer and she is engaged in volunteering activities related to that).*

#### 4.2.3. Emotional Changes

*Improved regulation of negative emotions:* Some of the long-term volunteers reported that engagement in volunteering helped them to achieve better emotional regulation skills. They were able to manage negative emotions well (*“Short temperedness has gone, in a way I am telling you what I was”; “My emotions are getting trained.”; “I have calmed down a lot. Initially I was extremely frustrated, so that has gone down—the overall level.”; “It takes away lot of depression…It takes away your anger, depression.”).*

*Increased frustration tolerance:* Ability to tolerate frustration about not getting something that one had wished for, or delaying the gratification of one’s desires was a change reported by one long-term volunteer *(“…my attitude has changed, because initially I use to get irritated for small things for example, if I like something and if I don’t get it, I used to feel…Even now, sometimes I wish for some things, but immediately the thought comes that there are people who don’t even have what we have. It calms me down”).*

*Increased experience of contentment and other positive emotions*: Several long-term volunteers spoke about a sense of contentment that was derived from engagement in volunteering *(e.g., “I have started thinking generally in life it is not good to expect too much…The quantum of craving has come down”; “Once I started going for volunteering, my attitude towards life has changed. My thinking that I don’t have anything, that was there, it changed. I have some rich friends and we are middle class family, so that longing for what we don’t have was there. That attitude has changed now. I feel more content”)*. This was one example wherein a cognitive change (attitudinal change) was linked to frequency of experiencing a positive emotion (contentment). One of the important contributors for satisfaction with volunteering was mentioned in terms of positive emotions, experienced during the volunteering. A few examples include a positive feeling derived from the responsiveness shown (“…the pleasure which you get when an animal is treated or fed…just their wagging of their tail you know it gives you all the joy for the day…”)/impact on the recipients as a result of the volunteer’s engagement in volunteering (“The response of the children. They are able to enjoy the class and apply what we are teaching in the class”) and a sense of fulfilling the commitment towards the organization/society.

### 4.3. Well-Being in Long-Term Volunteers

The Table 2 presents descriptive statistics of the measures used. All the measures had satisfactory internal consistency with alpha coefficients ranging between 0.81 and 0.88. The scores on the well-being indices (except self-acceptance subscale of psychological well-being) were fairly distributed and did not depart significantly from normal distribution. The mean scores on life satisfaction and psychological well-being were well above the mid points of the possible range of scores. On all the measures, higher scores reflect higher well-being except that on negative affect, higher scores reflect higher levels of negative affect and thus lower level of well-being.

### 4.4. Comparison with Short-Term Volunteers

The short-term volunteers were drawn from the larger study that had prospectively followed new volunteers who had volunteered for a six-month time frame. A sample of 21 short-term volunteers was drawn from the larger sample in such a way that it was comparable to the long-term volunteer sample on age, gender and broad income category. These two groups did not differ from each other on age (*t* = 1.20, *p* > 0.05), gender (χ^2^ = 0.59, *p* > 0.05 and broad income categories (χ^2^ = 2.52, *p* > 0.05). Their profile was broadly similar to the long-term volunteer sample. They were between 21 and 50 years, with their mean age being 29 years. The majority (*n* = 17) were young adults (18–35 years) and the remaining 4 were middle aged. There was a preponderance of females in the short-term volunteer sample too, with 14 females and 7 males and the majority (*n* = 15) were engaged in full time paid work. About two third had undergraduate level qualification. There was a small preponderance of unmarried participants (*n* = 13) and a majority were Hindu by religion (*n* = 16). This sample was drawn from the same pool of NGOs as the long-term volunteers and they were involved in multiple kinds of volunteering activities. The average age at which they initiated volunteering for a social cause was 23 years, the same as the long-term volunteers. While 13 had started volunteering between 18 and 28 years of age, 2 had started during childhood and early adolescence. In the past 6 months, almost half were volunteering on a more than once a week basis (*n* = 9). Others reported to be volunteering on a once a week or once in a 2–4 week basis. This sample also showed a similar pattern as compared to long-term volunteers in terms of quantum of volunteering per month.

Comparison of short-term volunteer sample with long-term volunteers revealed that the latter were significantly higher on overall psychological well-being and on three of its subscales, namely, self-acceptance, mastery and competence and engagement and growth, as compared to the short-term volunteers (Table 3).

## 5. Discussion

Sufficient heterogeneity in the long-term volunteers sample was successfully achieved through purposive sampling and recruitment of participants who were varied in terms of age, gender, marital status, educational level, volunteering duration as well as nature of volunteering work. Most of the long-term volunteers in the present study who initiated formal volunteering at an early age reported some form of early exposure such as being part of social service schemes (e.g., National Service Scheme-NSS) in schools/colleges. Exposure through students-wings of political parties and offering help in religious institutions (temples, churches, etc.) were also reported by a few long-term volunteers who opined that these provided a platform for them to become aware about social issues and volunteer at an early age. These observations point to the potential utility of such exposures at familial and societal levels for cultivating a pro-volunteering attitude from an early age and are in line with other studies [42,43]. The long-term volunteers in the present study not only had a long history of volunteering, but their level of engagement was also fairly high. The sample hence provided a useful opportunity to explore in-depth, the experience of volunteering in terms of its perceived benefits.

All the long-term volunteers reported a change in one or more of the three domains (cognitions, behaviors and emotions) as a result of volunteering. A majority expressed that through engagement in volunteering they were able to go beyond “self” and understand others’ perspectives. Volunteering helped them to strengthen some of the values or acquire new values (e.g., citizenship), as well as become more aware about the negative consequences of certain behaviors for the society in the long run. Other emergent themes were about learning how to ignore minor issues in personal life, gaining a sense of purpose in life, becoming clearer about life-goals, developing heightened awareness about the surroundings and in becoming more willing to go beyond comfort zones.

A significant number of long-term volunteers reported behavioral changes some of which were noted by the significant others too. These included decline in aggressive tendencies and interpersonal problems, and becoming more assertive for the social rights of people in general. Another important behavioral change reported in the present study sample was persistence in goal pursuit. Formal volunteering entails planned and sustained action [3]. Hence, engaging in regular volunteering may provide a means to practice self-regulation and bring more focus and persistence in actions, which can also be reflected in personal goal pursuit of an individual.

It is important to note that emotional benefits reported were not restricted to the generation of positive emotions and protection from negative emotions. Volunteering was seen as helpful in improving tolerance to frustrations and bringing a sense of contentment. This is in line with the observation that volunteering has actually been recommended as one of the important distress-tolerance activities in Dialectic Behavior Therapy, especially for people with borderline personality disorder [44].

The positive changes in beliefs, behaviors and emotions reported by the long-term volunteers are likely to underlie the high scores on psychological well-being subscales seen in the quantitative analysis. The positive effects noted on various subscales of psychological well-being (self-acceptance, sense of mastery and competence, sense of engagement and growth) to an extent mirrored the interview-based rich qualitative data. Gains in positive relations, sense of engagement and growth as well as self-acceptance, sense of mastery and competence (various dimensions of psychological well-being) may accrue as a result of changes in one’s perspective about minor personal issues, and interpersonal relationships, increased tolerance and empathy, self-regulation, frustration-tolerance and better management of negative emotions in general.

This study was cross-sectional in nature and the well-being scores reflected the current levels of well-being of the volunteers. Also, it is difficult to comment on the absolute scores on the psychological well-being measures in the long-term volunteers’ sample, due to a lack of directly comparable studies involving general community samples. As far as subjective well-being scores were concerned, the mean values of the long-term volunteers sample in the present study on positive affect and life-satisfaction were close to those reported in a large-scale Indian study utilizing a general community study with a wide age range of participants [36]. On the other hand, mean negative affect of long-term volunteers sample in the present study was much lower than that reported in Agrawal et al.’s study [36]. A longitudinal design as well as use of a matched non-volunteers sample would help in further understanding the relationship between long-term volunteering and well-being.

In the present study, well-being scores of long-term volunteers could be compared with a matched group of short-term volunteers. It was found that long-term volunteers had significantly higher overall psychological well-being and higher scores on three out of the four subscales of psychological well-being, namely, self-acceptance, sense of mastery and competence and sense of engagement and growth as compared to the short-term volunteers. This pattern extends the findings from previous research on the beneficial effects of continued volunteering on well-being [18,19]. The researcher did not come across any study that has assessed psychological well-being as an outcome of volunteering, with most studies having focused on emotional well-being outcomes. The present study results suggest that beneficial effects on psychological well-being may accrue over the course of volunteering engagement in the longer run.

The probable mechanisms through which various changes (as reported by the long-term volunteers) may have occurred needs to be explored in-depth. Some of the mechanisms that seemed to emerge in the long-term volunteers’ narratives were: an opportunity to interact and witness living conditions of deprived persons, which otherwise went unnoticed. Some volunteers used the term “witnessing the raw life”. This witnessing might have helped the volunteers in developing a sense of social connection through perspective-taking. Social connections are strongly correlated with well-being and positive social behaviors [45]. “A realization of being more fortunate than some others”, as mentioned by some participants, may also have played a role in their well-being. Research suggests that counting one’s blessings [22] and a sense of gratitude [46] are correlated with well-being. Development of compassion over time, an opportunity to observe different perspectives on a social issue [45], and a change in others’ perception about the volunteer and engagement in a meaningful activity per se [23] are a few other mechanisms that require exploration. The personal values espoused by the volunteers were not directly assessed in the present study. However, it is plausible that initiating volunteering in order to express altruistic and humanitarian values may help in maintenance/gains in well-being though pursuit of valued and meaningful goals in the form of volunteering.

Several of these self-reported changes also point towards enhancement of social well-being which was not directly or explicitly examined in the study. The concept of social well-being has been used to describe an individual’s perceptions of the quality of their relationships with other people, their neighborhoods and their communities [47]. It is the appraisal of one’s circumstance and functioning in society. From an Adlerian theoretical viewpoint, volunteering has components of “social interest”. Social interest is the sense of being part of the community and willingness to participate/contribute to society (Adler 1964 as cited in [48]. Development of social interest is considered as an important aspect of healthy personality and is indicated in achieving mental health [48]. Volunteering can be seen as one form of the behavioral aspect of social interest. The narratives about perceived-changes in personal attributes (especially cognitive and behavioral domains) suggest that long-term volunteering may have contributed to strengthening of social interest and social well-being. A few of the perceived changes, namely, enhanced awareness of repercussions of one’s behaviors and awareness of environmental issues, practice of civic duties, increased tolerance and empathy correspond to positive changes in sense of social contribution (perceiving one’s life as useful to the society) and social acceptance (having a positive attitude towards others) which are the dimensions of social well-being [47]. The overall study observations against a background of burgeoning literature on the benefits of volunteering suggest the potential powerful role of encouraging youth engagement in volunteering or in other words cultivating a pro-volunteering culture in a given society. Development of volunteering as a public health intervention has been discussed [15] as a potential measure to promote mental health and survival. We believe that it is likely to influence not merely mental health but also social wellbeing in those who sustain their volunteering engagement.

The study has several limitations. These include its cross-sectional nature, a small sample size and lack of information on the baseline characteristics of the participants before their initiation into formal volunteering. Inclusion of an additional matched sample of non-volunteers may have added further light on understanding the patterns of results; however, this could not be accomplished due to logistical constraints including the time-bound nature of the study. Its cross sectional nature places constraints on causal inferences though changes in self as described by long-term volunteers, and their elevated well-being scores as compared to a matched group of short-term volunteers do raise the hypothesis that long-term, formal volunteering can contribute to enhanced psychological and social well-being. The comparison of long-term volunteers with their short-term counterparts was done using standardized measures. It would be useful to qualitatively examine perceived changes in short-term volunteers too, in future studies. Owing to the small sample size, subgroup analyses pertaining to age and gender could not be carried out. Although inspection of the qualitative data did not suggest such differential patterns, these need to be carefully examined in larger samples. We also wish to reiterate that broad categorization of changes in cognitive, behavioral and emotional domains merely reflected the levels at which the changes were described by the participants and the examples shared reflected how changes in various domains tended to co-occur. The study relied on self-reports as it aimed to uncover the volunteers’ own perspectives. The possibility of social desirability response biases cannot be completely ruled out; however, attempts were made to minimize it through adopting a non-judgmental stance during the interviews, enquiring about all kinds of experiences as well as challenges, etc., and particularly through encouraging participants to elaborate the basis of their perceptions/illustrate using specific incidents and examples. In several instances, the participants spontaneously reported how the positive changes they were describing had been noticed and commented upon by their significant others, too.

## 6. Conclusions

This exploratory study has yielded rich insights about the links between sustained engagement in formal volunteering and various facets of well-being. The findings highlight the need for further research in the field using mixed method longitudinal designs. The study observations point towards the potential utility of initiatives or programs that provide exposure and easy access to volunteering opportunities as a public health intervention.

## Figures and Tables

**Table 1:** Perceived changes in self: Long-term volunteers (*n* = 20).

Serial Number	Broad Domains of Changes	Frequency
1	Cognitive	16
2	Behavioral	13
3	Emotional	9

**Table 2:** Descriptive statistics on well-being measures: Long-term volunteers.

Measure	Min-Max Scores Possible	Min-Max Scores Obtained	Mean (SD)	KS-Z Value (Normality Assessment)
Positive affect	13–65	21–65	44.75 (11.39)	0.71
Negative affect	13–65	13–41	19.90 (6.50)	0.78
Life satisfaction	5–35	12–33	26.30 (4.35)	0.82
Psychological well-being subscales				
Self-acceptance	4–24	16–24	21.95 (2.44)	1.38 *
Mastery & Competence	6–36	20–36	29.85 (4.13)	0.53
Positive Relations	5–30	11–30	25.10 (5.07)	0.75
Engagement & Growth	5–30	20–30	27.30 (2.92)	0.87
Overall Psychological well-being	20–120	76–120	104.20 (10.82)	0.64

* *p* < 0.05.

**Table 3:** Comparison of long-term and short-term volunteers on well-being indices.

Variable	Type of Volunteers				*t*-Value
	Long-Term Volunteers (*n* = 20)		Short-Term Volunteer Sample (*n* = 21)		
	Mean	SD	Mean	SD	
Positive affect	44.75	11.39	42.81	9.02	0.61
Negative affect	19.90	6.50	23.29	8.42	1.44
Life satisfaction	26.30	4.35	24.62	4.67	1.19
Psychological well-being	104.20	10.82	93.71	11.44	3.01 *
Psychological Well-being Subscales					
Self-acceptance	21.95	2.44	19.33	3.23	#2.84 *
Mastery & competence	29.85	4.13	25.38	5.00	3.11 *
Positive relations	25.10	5.07	24.57	4.93	0.34
Engagement & growth	27.30	2.92	24.43	3.25	2.97 *

# denotes Mann Whitney U Z value. Others are t-values; * *p* < 0.01 (2-tailed).

## References

[1] Smith D.B. (1998). Volunteer patterns of mid- and later life American couples. J. Sociol. Soc. Welf..

[2] Kitchen S., Michaelson J., Wood N., John P. (2006). 2005 Citizenship Survey.

[3] Penner L.A. (2002). Dispositional and organizational influences on sustained volunteerism: An interactionist perspective. J. Soc. Issues.

[4] Cherian M. (2012). Volunteering Among Faiths—The Religious Lexicon. Volunteering in India: Contexts, Perspectives and Discourses.

[5] Choudhury B.L., Shome B. (2012). Indian Perspective and the Tradition of Volunteering. Volunteering in India: Contexts, Perspectives and Discourses.

[6] Chatterjee P. (2006). The Changing Face of Volunteering in India. http://www.worldvolunteerweb.org/news-views/viewpoints/doc/the-changing-face-of.html.

[7] Anik L., Aknin L.B., Norton M.I., Dunn E.W. (2009). Feeling Good about Giving: The Benefits (and Costs) of Self-Interested Charitable Behavior.

[8] Wilson J., Musick M. (2000). The Effects of volunteering on the volunteer. Law Contemp. Probl..

[9] Roker D., Player K., Coleman J. (1998). Challenging the image: The involvement of young people with disabilities in volunteering and campaigning. Disabil. Soc..

[10] Swinson J.L. (2006). Focusing on the health benefits of volunteering as a recruitment strategy. Int. J. Volunt. Adm..

[11] Li Y., Ferraro K.F. (2006). Volunteering in middle and later life: Is health a benefit, barrier or both?. Soc. Forces.

[12] Thoits P.A., Hewitt L.N. (2001). Volunteer work and well-being. J. Health Soc. Behav..

[13] Meier S., Stutzer A. (2004). Is volunteering rewarding in itself?. IZA Discuss. Paper.

[14] Dass-Brailsford P., Thomley R., de Mendoza A.H. (2011). Paying it forward: The transformative aspects of volunteering after Hurricane Katrina. Traumatology.

[15] Jenkinson C.E., Dickens A.P., Jones K., Thompson-Coon J., Taylor R.S., Rogers M., Bambra C.L., Richards S.H. (2013). Is volunteering a public health intervention? A systematic review and meta-analysis of the health and survival of volunteers. BMC Publ. Health.

[16] Shen H.W., Pickard J.G., Johnson S.D. (2013). Self-esteem mediates the relationship between volunteering and depression for African American caregivers. J. Gerontol. Soc. Work.

[17] Tabassum F., Mohan J., Smith P. (2016). Association of volunteering with mental well-being: A lifecourse analysis of a national population-based longitudinal study in the UK. BMJ Open.

[18] Binder M., Freytag A. (2013). Volunteering, subjective well-being and public policy. J. Econ. Psychol..

[19] Musick M.A., Wilson J. (2003). Volunteering and depression: The role of psychological and social resources in different age groups. Soc. Sci. Med..

[20] Magen Z., Aharoni R. (1991). Adolescents’ contributing toward others: Relationship to positive experiences and transpersonal commitment. J. Humanist. Psychol..

[21] Emmons R.A., McCullough M.E. (2003). Counting blessings versus burdens: An experimental investigation of gratitude and subjective well-being in daily life. J. Personal. Soc. Psychol..

[22] Seligman M.E.P. (1991). Learned Optimism.

[23] Snyder M., Omoto A.M., Wosinska W., Cialdini R.B., Barrett D., Reykowski J. (2001). Basic research and practical problems: Volunteerism and the psychology of individual and collective action. The Practice of Social Influence in Multiple Cultures.

[24] Sheldon K.M., Ryan R.M., Reis H.T. (1996). What makes for a good day? Competence and autonomy in the day and the person. Personal. Soc. Psychol. B.

[25] Lerner R.M., Lopez S.J., Snyder C.R. (2009). The positive youth development perspective: Theoretical and empirical bases of a strengths-based approach to adolescent development. Oxford Handbook of Positive Psychology.

[26] Sheldon K.M., Lyubomirsky S., Linley A.S., Joseph S. (2004). Achieving Sustainable New Happiness: Prospects, Practices, and Prescriptions. Positive Psychology in Practice.

[27] Sheldon K.M., Lyubomirsky S. (2006). How to increase and sustain positive emotion: The effects of expressing gratitude and visualizing best possible selves. J. Posit. Psychol..

[28] Peterson C., Park N., Seligman M.E.P. (2005). Orientations to happiness and life satisfaction: The full life versus the empty life. J. Happiness Stud..

[29] Magoo G., Khanna R. (1991). Altruism and willingness to donate blood. J. Personal. Clin. Stud..

[30] Rai S.N., Gupta M.D. (1995). Donating behaviour as a function of age, culture and outcome feedback conditions. Psycho-Lingua.

[31] Bhagaokar R., Mehta D. (2012). Youth civic engagement in India: A case in point. Psychol. Dev. Soc. J..

[32] Parasuraman S. (2009). An Evaluation Study of NSS in India 2008–09.

[33] Watson D., Clark L.A., Tellegen A. (1988). Development and validation of brief measures of positive and negative affect: The PANAS scales. J. Personal. Soc. Psychol..

[34] Barett L.F., Russell J.A. (1998). Independence and bipolarity in the structure of current affect. J. Personal. Soc. Psychol..

[35] Rao D., Mehrotra S. (2006). Negotiation of life-tasks and subjective well-being in young adults pursuing professional courses. Psychol. Stud..

[36] Agarwal J., Murthy P., Philip M., Mehrotra S., Thennarasu K., John P.J., Girish N., Thippeswamy V., Isaac M. (2010). Socio-demographic correlates of subjective well-being in urban India. Soc. Indic Res..

[37] Diener E., Emmons R.A., Larsen R.J., Griffin S. (1985). The satisfaction with life scale. J. Personal. Assess.

[38] Mehrotra S., Tripathi R., Banu H. (2013). Psychological well-being: Reflections on an elusive construct and its assessment. J. Indian Acad. Appl. Psychol..

[39] Ryff C.D. (1989). Happiness is everything, or is it? Explorations on the meaning of psychological well-being. J. Personal. Soc. Psychol..

[40] Sandelowski M., Barroso J. (2003). Classifying the findings in qualitative studies. Qual. Health Res..

[41] Vaismoradi M., Turunen H., Bondas T. (2013). Content analysis and thematic analysis: Implications for conducting a qualitative descriptive study. Nurs. Health Sci..

[42] Janoski T., Musick M., Wilson J. (1998). Being volunteered? The impact of social participation and pro-social attitudes on volunteering. Sociol. Forum.

[43] Perry J., Brudney J., Coursey D., Littlepage L. (2008). What drives morally committed citizens? A study of the antecedents of public service motivation. Public Admin. Rev..

[44] Linehan M.M. (1993). Skills Training Manual for Treating Borderline Personality Disorder.

[45] Seppala E., Rossomando T., Doty J.R. (2013). Social connection and compassion: Important predictors of health and well-being. Soc. Res. Int. Q..

[46] Wood A.M., Froh J.J., Geraghty A.W. (2010). Gratitude and well-being: A review and theoretical integration. Clin. Psychol. Rev..

[47] Keyes C.L.M. (1998). Social well-being. Soc. Psychol. Q..

[48] Jones-Smith E. (2012). Theories of Counselling and Psychotherapy: An Integrative Approach.

